# Role of the endothelial reverse mode sodium-calcium exchanger in the dilation of the rat middle cerebral artery during hypoosmotic hyponatremia

**DOI:** 10.1007/s00424-022-02770-z

**Published:** 2022-11-17

**Authors:** Katarzyna Klapczyńska, Marta Aleksandrowicz, Ewa Koźniewska

**Affiliations:** 1 Institute of Physical Culture Sciences, Faculty of Health and Physical Education, University of Szczecin, Szczecin, Poland; 2 Laboratory of Experimental and Clinical Neurosurgery, Mossakowski Medical Research Institute Polish Academy of Sciences, Warsaw, Poland

**Keywords:** Hypoosmotic hyponatremia, Isolated middle cerebral artery, KB-R7943, Nitric oxide, Vasodilation

## Abstract

A decrease in serum sodium ion concentration below 135 mmol L^−1^ is usually accompanied by a decrease in plasma osmolality (hypoosmotic hyponatremia) and leads to the disorder of intracranial homeostasis mainly due to cellular swelling. Recently, using an in vitro model of hypoosmotic hyponatremia, we have found that a decrease in sodium ion concentration in the perfusate to 121 mmol L^−1^ relaxes the isolated rat middle cerebral artery (MCA). The aim of the present study was to explore the mechanism responsible for this relaxation. Isolated, pressurized, and perfused MCAs placed in a vessel chamber were subjected to a decrease in sodium ion concentration to 121 mmol L^−1^. Changes in the diameter of the vessels were monitored with a video camera. The removal of the endothelium and inhibition of nitric oxide-dependent signaling or the reverse mode sodium-calcium exchanger (NCX) were used to study the mechanism of the dilation of the vessel during hyponatremia. The dilation of the MCA (19 ± 5%, *p* < 0.005) in a low-sodium buffer was absent after removal of the endothelium or administration of the inhibitor of the reverse mode of sodium-calcium exchange and was reversed to constriction after the inhibition of nitric oxide (NO)/cGMP signaling. The dilation of the middle cerebral artery of the rat in a 121 mmol L^−^^1^ Na^+^ buffer depends on NO signaling and reverse mode of sodium-calcium exchange. These results suggest that constriction of large cerebral arteries with impaired NO-dependent signaling may be observed in response to hypoosmotic hyponatremia.

## Introduction

Sodium is the main osmotically active ion of the extracellular fluid. The physiological concentration of Na^+^ in the intracellular space is close to 10 mmol L^−1^, whereas the extracellular concentration is maintained at 144 mmol L^−1^. The difference in Na^+^ concentration across the cell membrane is essential for the maintenance of cell volume, resting membrane potential, and sodium-dependent transport systems [5]. Reduction in the extracellular concentration of Na^+^ generates an osmotic gradient across the cell membrane and leads to the inward movement of water and cellular swelling. Cells defend themselves against osmotic swelling by extrusion ions (K^+^ and Cl^−^) and organic osmolytes (myo-inositol, phosphocreatine and amino acids) to decrease intracellular osmolarity and volume [6, 13, 16, 35, 36]. This response, known as regulatory volume decrease (RVD), is observed in most mammalian cells studied in vitro including vascular endothelial cells [36].

It has been demonstrated by many research groups that, in a variety of cells types, RVD is preceded by an increase in the intracellular concentration of Ca^2+^ [4, 9, 10, 40]. The source of these calcium ions is uncertain. The influx of extracellular calcium and the release of calcium from intracellular stores or both have been proposed [4, 10, 40].

One of the possible mechanisms introducing Ca^2+^ into the cell under low extracellular sodium conditions could be an Na^+^/Ca^2+^ exchanger (NCX) which is present in the plasma membrane of virtually all cells [7, 37]. The activity and direction (forward or reverse) of the NCX is controlled mainly by a transplasmalemmal sodium gradient and membrane potential [18, 37]. In a physiological gradient of Na^+^ concentration across the cell membrane, NCX is considered to operate in the forward mode, i.e., transports one Ca^2+^ out of the cell in exchange for three Na^+^ entering the cell, thus participating in the intracellular homeostasis of calcium ions (e.g., cardiac muscle and neurons). A reduction in the extracellular concentration of sodium ions decreases the strength of the electrochemical gradient of these ions and may reverse the direction of NCX [23–25]. However, the reverse mode (Ca^2+^ entry) of the NCX has been reported to operate in a variety of cells also under physiological conditions [7, 37]. There is experimental evidence that in blood vessels, the Ca^2+^ entry mode of NCX contributes to pressure-induced myogenic constriction [8, 39], as well as to endothelium-dependent vasodilation [1, 41, 45].

Recently, we have found that lowering the extracellular concentration of Na^+^ to the level diagnosed in symptomatic patients with hyponatremia (121 mmol L^−1^) elicits dilation of the rat middle cerebral artery (MCA) [3]. In the present study, we sought to identify the possible mechanism responsible for this dilation. Our data demonstrates that the MCA dilates in a low-sodium environment in an endothelium- and nitric oxide (NO)/cGMP-dependent fashion. The increase in the concentration of Ca^2+^ required to activate endothelial cells seems to result from the activation of the reverse mode of NCX in the endothelium. In addition, when the NO/cGMP pathway is inhibited, constriction of the MCA in response to the lowering of sodium ion concentration is noted. The last observation suggests that the MCA with dysfunctional NO/cGMP signaling will constrict in response to hypoosmotic hyponatremia of a similar magnitude.

## Material and methods

The procedures described were approved by the IV Local Ethics Committee for the Care and Use of Laboratory Animals for Experimental Procedures, National Medicines Institute in Warsaw.

Male adult Wistar rats (270–310 g, *n* = 50) were provided by the Animal House of Mossakowski Medical Research Institute, PAS. The animals were anesthetized with 5% halothane in N_2_O/O_2_ (70%/30%) and decapitated. The brain was removed and immersed in cold (4 °C) a 3-(N-morpholino)propanesulfonic acid (MOPS)-buffered saline solution with 1% dialyzed bovine serum albumin (BSA) at pH 7.40.

### Isolation and mounting of the MCA

Middle cerebral arteries (*n* = 92) were isolated and mounted in the organ chamber according to the procedure as described [3]. Briefly, the MCA was dissected and cleaned of connective tissue. A 3 mm long segment was transferred to the organ chamber filled with the MOPS-buffered saline solution containing 1% dialyzed BSA, mounted between two glass micropipettes, and fixed. An inlet pipette was connected to a pressure-servo system. The MCA was perfused at a rate 100 µL/min with the MOPS-buffered saline solution containing 1% dialyzed BSA at an intraluminal pressure of 80 mm Hg. The extraluminal bath was replaced with a MOPS buffered saline solution without BSA, slowly heated to 37 °C, and exchanged at a rate of 20 mL/min with the help of a peristaltic pump (Masterflex, Cole Parmer, USA). The chamber was placed on the stage of an inverted light microscope (Olympus CKX41) equipped with a video camera and a monitor. The internal diameter of the vessel was measured directly from the screen and recorded for off-line analysis. During the equilibration period (1 h), the vessel slowly developed a myogenic tone (constriction by approximately 35% of the initial diameter). After this time, the response to increased potassium ion concentration (20 mmol L^−1^) in the extraluminal bath was tested. Vessels that did not develop a myogenic tone or responded with less than a 25% increase in the diameter to 20 mmol L^−1^ KCl were discarded.

### Experimental protocol

After the KCl reactivity test and 15 min equilibration period in the MOPS-buffered saline, the vessel was studied according to one of the following paradigms (Table 1). Series 0 was a time control study in which pressurized and KCl reactivity-tested MCA was maintained in a MOPS buffered physiological saline solution (Na^+^ = 145 mmol L^−1^) and continuously monitored for 180 min to find out whether the diameter is stable over time, which is needed to perform the longest of the experiments listed below. In series I, the intra- and extraluminal concentration of Na^+^ was decreased to 121 mmol L^−1^ for 180 min. Although the 60th minute was chosen as a reference time for the measurement of MCA diameter, the vessel was left in the low-sodium buffer up to 180 min to test the stability of the MCA diameter under this condition.

**Table 1:** List of the experimental series

Series	Paradigm	MCA diameter
0	145 mmol L^−1^ Na^+^ for 180 min	No change
I	121 mmol L^−1^ Na^+^ for 180 min	Stable dilation
II	121 mmol L^−1^ Na^+^ + NMDG 121 mmol L^−1^ Na^+^ + Tris–HCl	Dilation Dilation
III	–Endo + 121 mmol L^−1^ Na^+^	No change
IV	L-name + 121 mmol L^−1^ Na^+^	Constriction
V	ODQ + 121 mmol L^−1^ Na^+^	Constriction
VI	121 mmol L^−1^ Na^+^ + KB-R7943	Dilation or no change depending on the dose
VII	121 mmol L^−1^ Na^+^ + BAPTA-AM	Dilation

Induction of hyponatremia by lowering the concentration of NaCl by 24 mmol L^−1^ resulted in a decrease in osmolality from 300 to 268 ± 5 mOsm kg^−1^, and a decrease in the concentration of Cl^−^ ions by 24 mmol L^−1^. Therefore, in series II, the intra- and extraluminal concentration of Na^+^ was lowered similarly as in series I, but the osmolality of the buffer was maintained at 300 ± 3 mOsm kg^−1^ either with N-methyl-D-glucamine (NMDG) or Tris–HCl. In the case of the Tris–HCl buffer, 24 mmol L^−1^ HCl was used to normalize the Cl^−^ ion concentration in the extracellular space to test if a deficit in Cl^−^ affects the response of the MCA to hyponatremia in our model. In the remaining series of experiments, the composition of the MOPS buffered saline solution was similar as in series I and the buffer was hypoosmotic (268 ± 5 mOsm kg^−1^). In series III, the participation of endothelial cells in the response of MCA to lowering of Na^+^ concentration to 121 mmol L^−1^ was investigated. Endothelial cells were removed before decreasing Na^+^ concentration by blowing 8 mL of air through the lumen of the vessel for 15 min. The effectiveness of endothelium removal was confirmed by the absence of dilation in response to the intraluminal administration of acetylcholine (ACh, 100 µmol L^−1^). Next, the response to the lowering of Na^+^ concentration was studied similarly as in series I. In series IV and V, the participation of nitric oxide (NO)/cyclic guanosine monophosphate (cGMP) signaling pathway in the response of the MCA to 121 mmol L^−1^ Na^+^ was tested. To inhibit NO production, 30 min before the lowering of Na^+^ concentration, a nonselective NO synthase (NOS) inhibitor, N^G^-nitro-L-arginine methyl ester (L-NAME, 10 µmol L^−1^), was added to the extravascular perfusate. The synthesis of cGMP was inhibited by the administration of the compound 1H-[1,2,4]oxadiozolo[4,3-a]quinoxalin-1-one (ODQ, 5 µmol L^−1^) to the extravascular perfusate 30 min before the lowering of Na^+^ concentration. Series VI was designed to study the participation of the reverse mode of the Na^+^/Ca^2+^ exchanger in the response of the MCA to low sodium environment. In this series, (2-[2-[4-(4-nitrobenzyloxy)phenyl] ethyl isothiourea mesylate (KB-R7943, 1 or 10 µmol L^−1^) was administered to the normal sodium MOPS-buffered saline solution prior to the lowering of sodium ion concentration and maintained throughout the observation time. The lower dose, 1 µmol L^−1^ KB-R7943, has been used to inhibit nonselective mechanosensitive cation channels (TRPC) in order to find out if these channels can provide a route for Ca^2+^ influx during hyponatremia in our experiments. It has been shown that KB-R7943 blocks TRPC at submicromolar concentrations, whereas a half-maximal block of the Ca^2+^ entry mode of the NCX is between 1 and 5 µmol L^−1^ [27]. The higher dose, 10 µmol L^−1^, has been used to inhibit the Ca^2+^ entry mode of the Na^+^/Ca^2+^ exchanger [12, 43].

In series VII, 1,2-bis(2-aminophenoxy)ethane-N,N,N′,N′-tetraacetic acid tetrakis(acetoxymethyl ester), (BAPTA-AM, 10 µmol L^−1^), a selective membrane permeable chelator of intracellular Ca^2+^, was administered intraluminally to the normal sodium MOPS-buffered saline solution prior to the lowering of sodium ion concentration and maintained throughout the observation time. At the dose 10 µmol L^−1^, BAPTA-AM fully buffers the intracellular Ca^2+^ in endothelial cells and minimally chelates Ca^2+^ in smooth muscle cells [49]. The aim of this experiment was to assess the contribution of endothelial Ca^2+^ to the response of the MCA to hypoosmotic hyponatremia.

### Drugs and solutions

The normonatremic MOPS buffered saline solution contained (in mmol L^−1^) 144.0 NaCl, 3.0 KCl, 2.5 CaCl_2_, 1.5 MgSO_4_, 1.21 NaH_2_PO_4_, 0.02 EDTA, 2.0 pyruvate, 5.0 glucose, and 2.0 MOPS. The concentration of solutes in the hyponatremic MOPS buffer was the same as in the normonatremic one except for NaCl, which was lowered to 120 mmol L^−1^ to obtain a Na^+^ concentration in the buffer equal to 121 mmol L^−1^. The hyponatremic MOPS buffer was hypoosmotic in all but one series (series II) of the experiments in which the osmotic pressure was maintained at 300 ± 3 mOsm kg^−1^ as mentioned above. Acetylcholine chloride, NMDG, L-NAME, BAPTA-AM, Tris, and nimodipine were purchased from Sigma-Aldrich. ODQ and KB-R7943 mesylate were obtained from Tocris Cookson Ltd. The drugs were dissolved in a MOPS buffered saline solution except for ODQ, BAPTA-AM, and KB-R7943 mesylate, which were prepared in DMSO and added to the buffer. The final concentration of DMSO in the MOPS buffered saline did not exceed 0.005%. According to the results of a pilot study, this concentration of DMSO does not affect the diameter of the MCA.

### Data analysis

All the data is presented as mean ± standard error (S.E.), and *n* denotes the total number of rats used or the number of vessels per group. The differences between the groups were assessed using one-way analysis of variance (ANOVA) followed by post hoc Tukey’s comparison (Statistica 10). Single comparisons were made using Student’s *t*-test for paired or unpaired data when appropriate. The differences were considered statistically significant at *p* < 0.05.

## Results

### Stability of the MCA diameter in normonatremic buffer

The results of time control series 0 demonstrate that during the observation time of 180 min the diameter of pressurized and perfused MCA maintained at 37 °C and pH = 7.4 (Table 2: normonatremia, time 0 min) remains stable (Table 2: normonatremia, time 60, 120, and 180 min).

**Table 2:** Stability of the MCA diameter in series 0 and I

Time (min)	MCA diameter (µm)	
	145 mmol L^−1^ Na^+^	121 mmol L^−1^ Na^+^
0	139 ± 8	165 ± 3
60	145 ± 13	162 ± 7
120	139 ± 4	167 ± 7
180	145 ± 5	168 ± 10

### Response of the MCA to 121 mmol L^−1^ Na^+^. Lack of the effect of hypotonicity

**Fig. 1:**
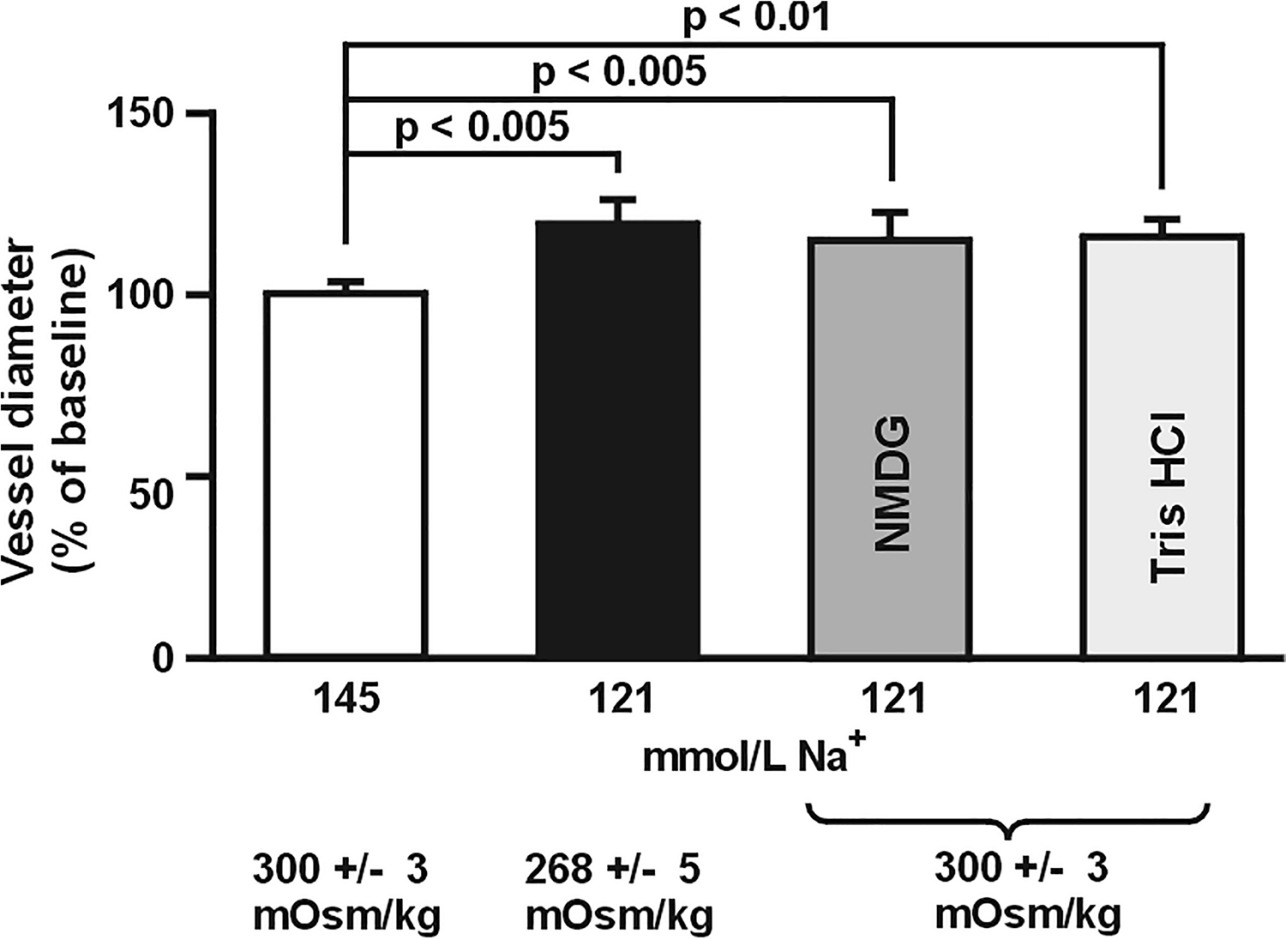
The middle cerebral artery dilates after the decrease in intra- and extravascular sodium ion concentration from 145 to 121 mmol L^−^^1^ without correction for the decreased osmolality (black bar). The correction of osmolality with N-methyl D-glucamine (NMDG) or Tris–HCl does not affect the response of the vessel to lowering of intra- and extraluminal sodium ion concentrations. Values are mean ± S.E. The number of vessels per group is between 6 and 8

Replacement of the normonatremic MOPS buffered saline solution with the one containing 121 mmol L^−1^ Na^+^ led to the dilation of the MCA beginning at 15 min after the induction of hyponatremia. At 60 min, the diameter of the MCA increased by 19 ± 5% (from 143 ± 8 to 169 ± 7 µm, *p* < 0.005) of the normonatremic control (Fig. 1 and Table 2: hyponatremia, time 0 vs. time 60 min) and remained at this level until the end of the observation time (Table 2: hyponatremia, time 60, 120, and 180 min). This response was solely dependent on the decrease in Na^+^ concentration in the buffer and not on the hypotonicity, or on a decrease in Cl^−^ concentration since normalization of osmolality with either N-methyl-D-glucamine (NMDG) or the Tris–HCl buffer did not affect the response (Fig. 1).

### Effects of the removal of endothelium and inhibition of NO/cGMP signaling pathway on the response of the MCA to 121 mmol L^−1^ Na^+^

**Fig. 2:**
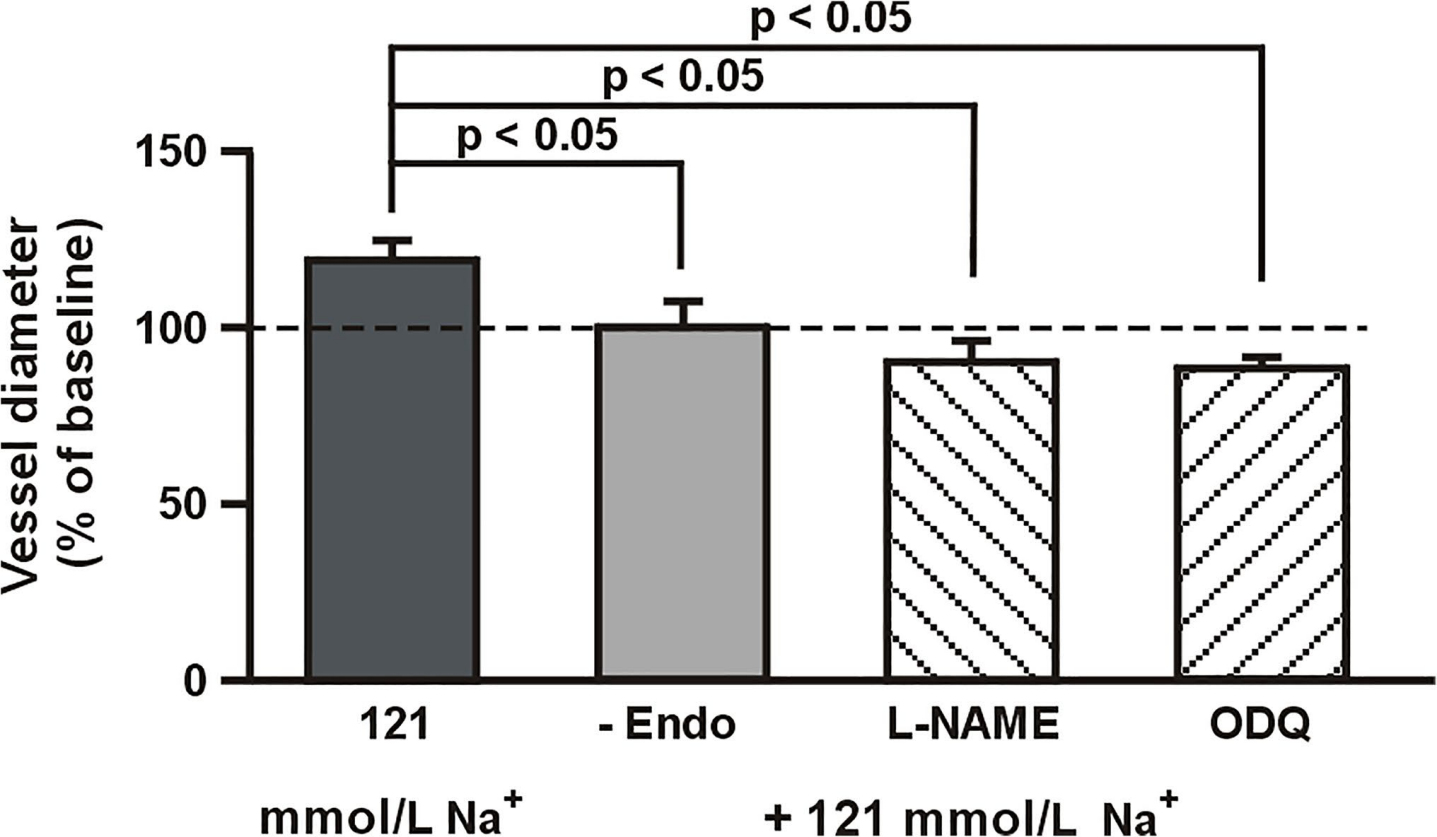
Removal of the endothelium (-Endo), inhibition of the synthesis of nitric oxide (L-NAME, 10 µmol L^−1^), or inhibition of guanylyl cyclase (ODQ, 5 µmol L^−1^) abolishes the dilation of the middle cerebral artery in the low-sodium buffer. Vessels pretreated with L-NAME or ODQ constricted during the lowering of Na^+^ to 121 mmol L^−1^ by 10 ± 5% (*p* < 0.05) and 12 ± 2% (*p* < 0.05), respectively. The horizontal line represents the reference diameter of the vessel during normonatremia, set at 100%. Values are mean ± S.E. The number of vessels per group is between 6 and 8

The successful removal of the endothelium was confirmed with 100 µmol L^−1^ acetylcholine, which did not affect the diameter of the MCA when applied intravascularly, in contrast to the 34 ± 13% dilation (*p* < 0.05) observed in endothelium-intact vessels. The diameter of the MCA without endothelium did not change when a 121-mmol L^−1^ Na^+^ MOPS-buffered saline solution was applied intra- and extraluminally (Fig. 2), suggesting that the endothelium-dependent vasodilator was responsible for the dilation of the MCA observed in the previous series. Accordingly, in the next series of experiments, the MCA was pretreated with the extraluminal addition of a nonselective NOS inhibitor, L-NAME (10 µmol L^−1^). This dose of L-NAME induced a 25 ± 6% constriction (from 152 ± 11 to 114 ± 13 µm, *p* < 0.05) during normonatremia. During the subsequent lowering of Na^+^ concentration to 121 mmol L^−1^, the MCA constricted by an additional 10 ± 2% (*p* < 0.05). When ODQ (5 µmol L^−1^), an inhibitor of the synthesis of cGMP, was administered during normonatremia, the MCA constricted by 35 ± 7% (from 145 ± 10 to 94 ± 7 µm, *p* < 0.02). The low-sodium buffer administered on the background of ODQ induced a further constriction of the vessel by 12 ± 5% (*p* < 0.05).

### Role of the reverse mode of Na^+^/Ca^2+^ exchanger in the response of the MCA to 121 mmol L^−1^ Na^+^

The addition of KB-R7943 to the normal sodium buffer prior to the lowering of Na^+^ concentration resulted in the stable and comparable dilation of the MCA by, on average, 23% (*p* < 0.02) in response to 1 µmol L^−1^ and 19% (*p* < 0.02) in response to 10 µmol L^−1^ KB-R7943. These diameters were considered new baselines.

**Fig. 3:**
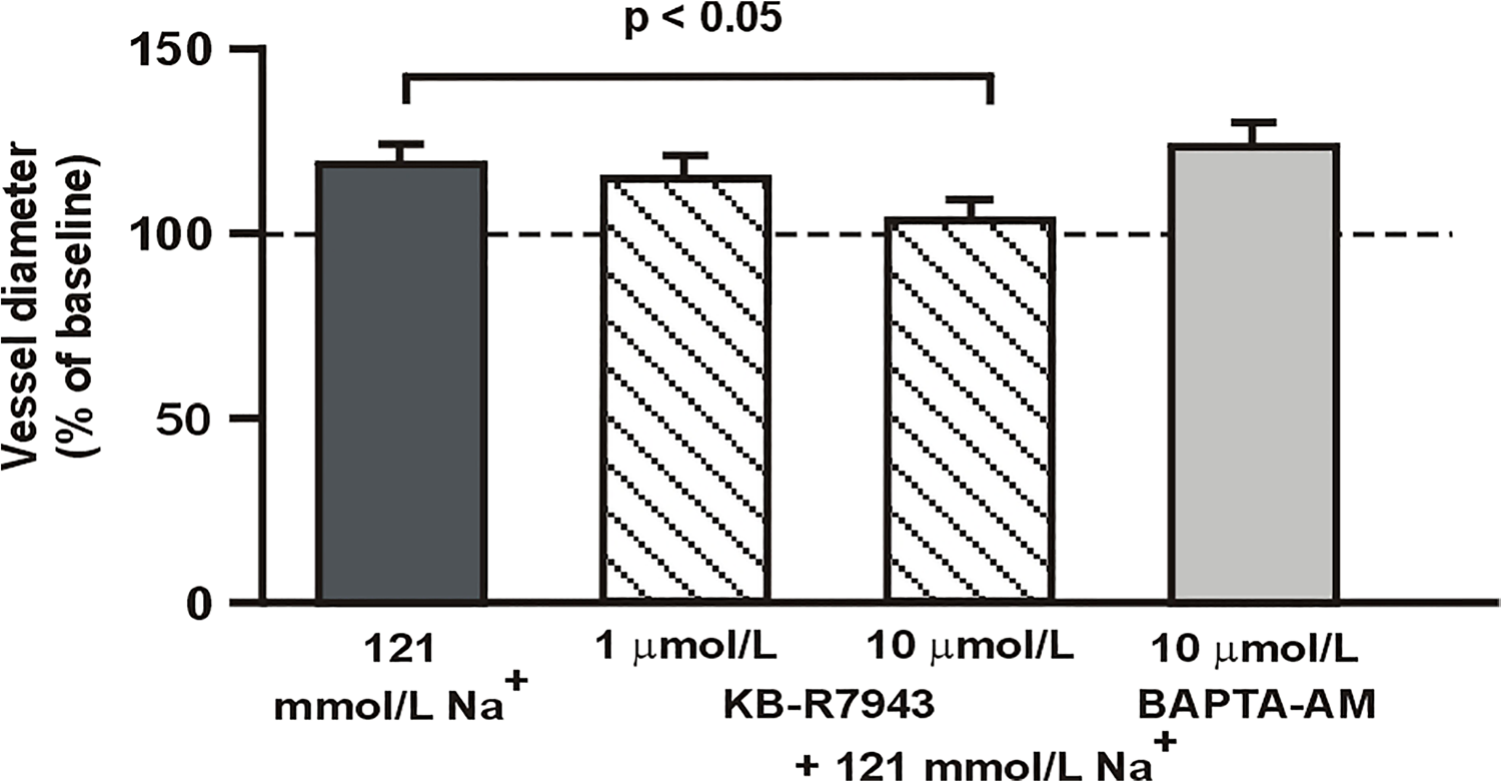
Administration of 1 µmol L^−1^ KR-R7943 did not affect the response of the MCA to low sodium buffer, whereas inhibition of the reverse mode of Na^+^/Ca^2+^ exchanger with 10 µmol L^−1^ KR-R7943 abolished the dilation of the MCA observed under low sodium conditions. Buffering intracellular Ca^2+^ in the endothelial cells with 10 µmol L^−1^ BAPTA-AM did not affect the response of the MCA to low sodium buffer. The horizontal line represents the reference diameter of the vessel during normonatremia, set at 100%. Values are mean ± S.E. The number of vessels per group is between 6 and 8

In the 121 mmol L^−1^ Na^+^ MOPS buffer containing 1 µmol L^−1^ KB-R7943, the MCA dilated by 15 ± 5%, which was not different from the response in the control group (Fig. 3). However, in the low sodium buffer containing 10 µmol L^−1^ KB-R7943, the diameter of the MCA did not change statistically significantly from the baseline.

To assess the possible role of the intracellular Ca^2+^ in the relaxation of the MCA in response to decreased Na^+^ concentration in the extracellular space, the chelator of the intracellular Ca^2+^ was administered intraluminally. The administration of BAPTA-AM (10 µmol L^−1^) to the normonatremic buffer resulted in the constriction of the MCA by 15 ± 2% (*p* < 0.01). In the 121-mmol L^−1^ Na^+^ MOPS buffer with BAPTA-AM, the MCA dilated by 26 ± 6% (*p* < 0.05), which was not statistically different from the response observed in the control series I, i.e., BAPTA-AM did not affect the response of the MCA to decreased Na^+^ concentration.

## Discussion

We have demonstrated that the dilatation of the isolated, pressurized, and perfused rat middle cerebral artery in response to the lowering of Na^+^ concentration in the perfusate i) depends on the lowering of extracellular sodium ion concentration and not on the decrease in osmolality and ii) requires the presence of an intact endothelium and nitric oxide/cGMP signaling. Our results suggest that the concentration of intracellular calcium ions required for the stimulation of the synthesis of nitric oxide by endothelial cells increases in a low sodium environment due to the activation of the reverse mode of the sodium/calcium exchanger. To the best of our knowledge, this is the first study that demonstrates normal cerebral blood vessels relaxing response to the lowering of extracellular sodium ion concentration through an endothelium- and NO/cGMP-dependent mechanism.

It is well established that extravascular osmolarity affects the tone of blood vessels. In hypoosmotic conditions, they constrict, whereas in a hyperosmotic environment, they relax [48]. Local increase in tissue osmolarity is considered to be one of the factors mediating functional hyperemia. It should be stressed, however, that our study addresses the problem of systemic decrease in sodium ion concentration. Also, in the present study, the effect of low sodium on the diameter of the MCA did not depend on hypoosmolarity as the MCA dilated in a normoosmotic low sodium buffer to the same extent as in the hypoosmotic one. The relaxation of the MCA in the low sodium environment observed in our study is endothelium-and NO/cGMP-dependent, since the removal of the endothelium and inhibition of NO/cGMP signaling abolish this response. In support of our data, it has been demonstrated that endothelial cells behave as vascular salt sensors and release increased amounts of NO when extracellular sodium ion concentration decreases [32]. It is important to stress that when the NO/cGMP system was inhibited, constriction of the MCA in the low sodium buffer was observed in our study instead of dilation.

NO is the most prominent endothelium-dependent vasodilator, produced with the participation of a specific endothelial synthase, eNOS, and released from the endothelium to activate soluble guanylate cyclase in the vascular smooth muscle [11]. The essential role of endothelial NO in physiological regulation of the cerebral vasculature is well recognized [19, 26]. It is also well established that activation of constitutive eNOS depends on increase in intracellular Ca^2+^ concentration. The increase in the intracellular concentration of Ca^2+^ in the low sodium/hypoosmotic buffer may be related to cell swelling (RVD does not completely restore the volume) and opening of stretch-activated Ca^2+^ channels such as, e.g., nonselective cation transient receptor potential (TRPC) membrane channels [47]. Our present data demonstrate that endothelium-dependent dilation of the MCA in a low sodium buffer is eliminated after inhibition of the reverse mode of NCX, which strongly suggests that the reverse mode of endothelial NCX operates during our experimental conditions. The reversal of the direction of NCX during the reduction in the extracellular concentration of sodium ions has been described in the literature [23, 24]. The participation of the reverse mode of NCX in physiological regulation of blood vessels is just emerging [8, 23, 39, 41, 44, 45, 52]. The reverse mode of NCX has been reported to contribute to the adjustment of the diameter of the posterior cerebral artery to perfusion pressure changes [23] and to the myogenic constriction of rat cremaster muscle first order arterioles [39]. According to Zhang [50] who reviewed the literature on the contribution of arterial NCX to the physiological regulation of myogenic tone, in pressurized arteries with myogenic tone and arteries in vivo, NCX is likely to operate in the “Ca^2+^ influx” mode. The present study shows for the first time that the “Ca^2+^ influx” mode of NCX participates in maintenance of the myogenic tone of isolated middle cerebral artery, since NCX inhibitor KB-R7943 administered at a dose of 10 µmol L^−1^, known to block the Ca^2+^ entry mode of the exchanger [22, 30, 44, 50, 52], resulted in the dilation of this vessel in normonatremia. Our study also indicates that TRPC plays a role in maintenance of the basal tone of isolated middle cerebral artery, as the MCA also dilated after administration of 1 µmol L^−1^ KB-R7943 in normonatremia. This lower dose of KB-R7943 was chosen by us to block TRPC channels based on the publication by Kraft who showed, as already mentioned, that half maximal inhibitory concentration (IC50) to block TRPC is below micromolar concentrations, whereas the IC50 of the Ca^2+^ entry mode of NCX is between 1 and 5 µmol L^−1^ [27].

It has been suggested that the reverse mode of NCX is involved in activation of endothelial NOS and release of NO by rat aortic endothelial cells [41], porcine aortic endothelial cells [45], and cardiac microvascular endothelial cells [24]. According to the results obtained by Bondarenko [8], NCX operates in the reverse mode in intact endothelial cells following stimulation by acetylcholine. Our present results strongly suggest that the activation of the reverse mode of the Na^+^/Ca^2**+**^ exchanger caused by decrease in the Na^+^ concentration gradient across the cell membrane also participates in the transport of Ca^2+^ into the endothelium. They also suggest that the reverse mode of the neuronal isoform of NCX is activated during lowering of Na^+^ concentration in the perfusate and participates in NO/cGMP-dependent dilation of the middle cerebral artery in response to hyponatremia. This suggestion is legitimate on the basis of the observation that removal of the endothelium eliminates dilation of the MCA in response to hyponatremia, whereas inhibition of NOS-dependent signaling with a nonselective NOS inhibitor (L-NAME blocks both constitutive NO synthases, endothelial eNOS and neuronal nNOS) or blockade of cGMP synthesis leads to MCA constriction in hyponatremia. This discrepancy may be best explained by the stimulation of both eNOS and nNOS in response to activation of the reverse-mode NCX. The more so, presence of functional nitrergic nerve fibers in the isolated rat MCA has been well documented [20, 31].

Another possible route of Ca^2+^ entry to the endothelium or nitrergic nerve endings during hyponatremia is the abovementioned TRP channels. These channels are commonly found in cellular membranes, participate in maintenance of basal myogenic tone, and as is also confirmed by our current data, are sensitive to stretch and participate in the dilation of cerebral blood vessels [21, 38, 51]. Our data do not confirm the participation of these channels in the influx of Ca^2+^ to cells in response to hyponatremia. KB-R7943, administered at a dose which blocks both the reverse mode of NCX and TPRC, eliminated the dilation of the MCA in response to hyponatremia, whereas inhibition of TPRC using a lower dose of KB-R7943 did not affect this response. However, further studies using well established TRPC inhibitors are needed to confirm this result. On the other hand, the experimental data published by some groups strongly suggest that the reverse mode of NCX is ideally situated in the cell membrane to deliver Ca^2+^ for the activation NOS. It has been shown that the Ca^2+^ influx associated with activation of the reverse mode of NCX does not lead to global increase in intracellular concentration of Ca^2+^ but occurs locally in the subplasmalemmal domain [45]. The important finding is also that the NCX1 protein (endothelial isoform of NCX) is detected in caveolin-rich membrane fractions containing both eNOS and caveolin-1, and that eNOS and NCX1 are located in close spatial proximity to each other [30, 45].

Our results showing no effect of the buffering of intracellular Ca^2+^ in the endothelium on the response of the MCA to hyponatremia strongly support the extracellular origin of the calcium signal required to activate NOS. The administration of BAPTA-AM at a dose which fully buffers the intracellular Ca^2+^ in endothelial cells and minimally chelates the Ca^2+^ in smooth muscle cells [49] did not affect the MCA response to lowering of extracellular Na^+^ concentration. Thus, intracellular Ca^2+^ does not participate in the response of the MCA to hyponatremia.

The results of our experiment with BAPTA-AM and the observations by others that during activation of the reverse mode of NCX in the endothelium intracellular Ca^2+^ concentration does not increase limits the possibility of participation of endothelial Ca^2+^-activated hyperpolarizing K^+^ currents such as IK_Ca_ and SK_Ca_ in the response of the MCA to hyponatremia [17]. However, the participation of hyperpolarization of the endothelium, which may propagate through myoendothelial gap junctions to hyperpolarize smooth muscle cells, cannot be excluded as an additional mechanism of hyponatremia-evoked dilation of the MCA [42]. The reductions in the extracellular concentration of NaCl may shift the membrane potential of vascular cells toward a more hyperpolarized state. Although the dilation of the MCA in the hyponatremic buffer in the experiment with administration of Tris–HCl and normalization of osmolality and chloride ion concentration was similar as in the control group, the hyperpolarization of endothelial cells during hyponatremia cannot be excluded. The direct hyperpolarization of smooth muscle cells during hyponatremia is, however, less probable as there is no dilation of the MCA in response to hyponatremia when the endothelium is removed.

Although there are data in the literature questioning KB-R7943 selectivity, they do not apply to the endothelial aspect of our study. It has been demonstrated that, in addition to the inhibition of the reverse mode of NCX, KB-R7943 may block the voltage-dependent, L-type Ca^2+^ channel of the cell membrane [33]. However, the dilation of the MCA in the low-sodium buffer in our experiments is entirely endothelium-dependent and the L-type voltage-dependent Ca^2+^ channel does not seem to be present in the endothelium of the rat MCA [28]. Another study questioning the selectivity of KB-R7943 demonstrated that, in cultured human umbilical vein endothelial cells and freshly isolated smooth muscle cells of the mouse aorta, KB-R7943 not only inhibited the reverse mode of NCX but also activated the large conductance Ca^2+^-activated K^+^ channel (BK_Ca_) [29]. This argument also does not apply to our research, because these channels are irrelevant in the endothelium of cerebral blood vessels [2].

One may also question the use of NMDG to compensate for the osmolality in our study, as it was shown to activate outward currents [46], which, in vascular smooth muscles, could lead to hyperpolarization and relaxation. In order to avoid such argument, we also used Tris–HCl to normalize osmolality and Cl^−^ concentration. The result of this experiment confirmed that decrease in osmolality is irrelevant to the response of the MCA to hyponatremia.

Our results strongly suggest that reverse mode of NCX can be stimulated in endothelial cells of intact, isolated large cerebral arteries by a 24 mmol L^−1^ decrease in plasma sodium concentration. This, in turn, suggests that endothelial NCX is much more sensitive to decrease in Na^+^ gradient across the plasma membrane than the one operating in smooth muscle cells. The elucidation of this issue requires a further study.

In summary, these results demonstrate for the first time that during hypoosmotic hyponatremia, normal cerebral blood vessels dilate through an endothelium- and NO/cGMP-dependent mechanism. They also suggest that stimulation of the NO/cGMP signaling pathway in the middle cerebral artery of the rat during short-lasting lowering of the extracellular sodium concentration is associated with activation of the “Ca^2+^ entry” mode of the Na^+^/Ca^2+^ exchanger in the endothelium.

Last but not least, these results demonstrate that when NO-dependent regulation of the middle cerebral artery is impaired, the vessel constricts in response to the lowering of Na^+^ concentration in the extracellular fluid.

However, it should be stressed that the experiments reported here were performed on the middle cerebral artery of male rats. Taking into consideration the reported sexual differences in the response to hyponatremia and protective effects of estrogens on the regulation of cerebral blood vessels [14, 15, 34], a complete picture of the response to hyponatremia requires experiments on the middle cerebral artery of the female brain.

## Data Availability

All data that support the finding of the current study are available from corresponding author upon reasonable request.
